# The Role of miRNA in Regulating the Fate of Monocytes in Health and Cancer

**DOI:** 10.3390/biom12010100

**Published:** 2022-01-07

**Authors:** Anna Alwani, Aneta Andreasik, Rafał Szatanek, Maciej Siedlar, Monika Baj-Krzyworzeka

**Affiliations:** Department of Clinical Immunology, Institute of Paediatrics, Jagiellonian University Medical College, 30-663 Kraków, Poland; anna.alwani@doctoral.uj.edu.pl (A.A.); anetaandre@o2.pl (A.A.); rafal.szatanek@uj.edu.pl (R.S.); misiedla@cyf-kr.edu.pl (M.S.)

**Keywords:** monocytes, macrophages, miRNA, cancer

## Abstract

Monocytes represent a heterogeneous population of blood cells that provide a link between innate and adaptive immunity. The unique potential of monocytes as both precursors (e.g., of macrophages) and effector cells (as phagocytes or cytotoxic cells) makes them an interesting research and therapeutic target. At the site of a tumor, monocytes/macrophages constitute a major population of infiltrating leukocytes and, depending on the type of tumor, may play a dual role as either a bad or good indicator for cancer recovery. The functional activity of monocytes and macrophages derived from them is tightly regulated at the transcriptional and post-transcriptional level. This review summarizes the current understanding of the role of small regulatory miRNA in monocyte formation, maturation and function in health and cancer development. Additionally, signatures of miRNA-based monocyte subsets and the influence of exogenous miRNA generated in the tumor environment on the function of monocytes are discussed.

## 1. First Glance at Monocytes

Monocytes are highly plastic cells that link innate and adaptive immunity. In humans, they usually constitute less than 10% of all leukocytes [1] and are the largest white cells in the blood, measuring between 16 and 22 μm in diameter. Monocytes contain one large, kidney-shaped nucleus located in the center of the cytoplasm. They originate in the bone marrow from pluripotent hematopoietic stem cells via a series of progenitor cells. Finally, after the divisions of promonocytes, monocytes are formed and enter the circulation. Monocytes’ residence in the human blood lasts from 1 to approximately 7 days [2,3,4]. Circulating monocytes are a heterogenous population of cells (according to size, morphology, etc.) divided into subpopulations according to differences in the expression of surface markers, CD14 and CD16 in humans and Ly6C, CCR2, and CX_3_CR1 in mice [5]. Gene expression patterns depicted in human monocyte subsets confirm their common origin [6]. Monocytes are recruited immediately to the infected, injured, or cancerous tissue, where they differentiate into macrophages. Monocytes are unique cells with the potential to be precursor (e.g., of macrophages) and effector (as phagocytes or cytotoxic cells) cells. The current theory about macrophage origin considers their embryonic precursors and monocytes’ potential to reconstitute the macrophage pool depending on the time and tissue niche [7]. At the tumor site, monocytes compose a major population of infiltrating leukocytes. After reaching the neoplastic tissue, they differentiate into tumor associated macrophages (TAM), which, depending on the type of tumor, are either bad or good indicators of cancer treatment. The tremendous plasticity of monocytes and macrophages is tightly regulated at both the transcriptional and translational levels. This review summarizes the current understanding of the role of small regulatory miRNA in monocyte formation, maturation and function in health and cancer development.

## 2. Role of miRNA in Monocyte Development

Monocytes are generated from stem cells (HSC, hematopoietic stem cell) in the bone marrow via four intermediate maturational stages: multipotent progenitor (MPP), common myeloid progenitor (CMP), granulocyte-macrophage progenitor (GMP) and macrophage progenitor (MP). These stages are regulated at the transcriptional level mainly by C/EBPα (CCAAT/enhancer-binding protein α), PU.1 (Purine-rich box 1), IRF8 (IFN-regulatory factor 8) and RUNX1 (Runt-related transcription factor 1) factors [8].

**Figure 1:**
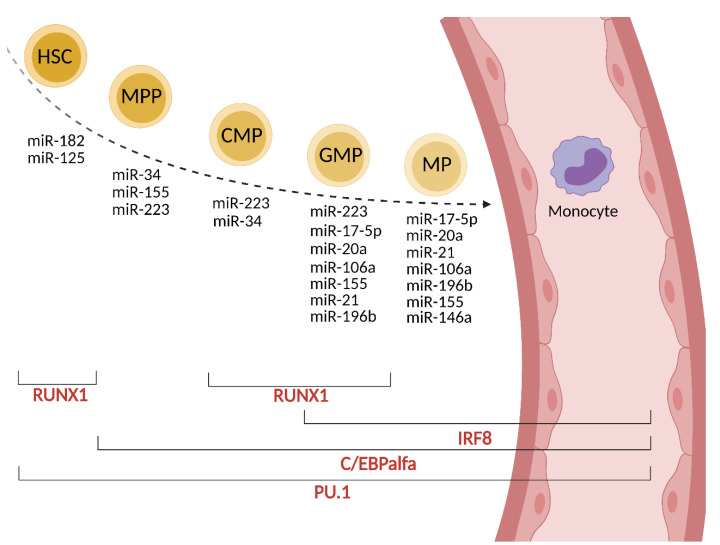
miRNA and transcription factors involved in monocyte formation.

In MPP cells, C/EBPα is upregulated and its activity is controlled by miR-182 and miR-34. C/EBPα directly upregulates the expression of miR-223 and miR-34, which are crucial for the formation of CMP [8]. The expression of miR-223 is repressed by nuclear factor I-A (NFI-A), which is a target for miR-223 (autoregulatory circuit). miR-223 is involved in the regulation of granulocytic maturation; whereas the lack of miR-223 promotes abundant granulopoiesis, its presence at higher levels promotes monopoiesis. PU.1 and C/EBPα are well-studied targets of miR-155 [9]. In turn, PU.1 upregulates the expression of miR-223, miR-146a, miR-155, and miR-338 [9]. The enhanced level of PU.1 promotes the maturation of GMP. IRF8 and RUNX1 are other transcription factors involved in normal myelopoiesis in cooperation with PU.1 [10]. PU.1 induces IRF8 expression, which further promotes monocyte over granulocyte differentiation potential in progenitors. RUNX1 is involved in the up-or down-regulation of miR-223 and, in turn, is down-regulated by miR-129 [11]. RUNX1 enhances the expression of the CSFR1 (M-CSFR, CD115) receptor for M-CSF, an important regulator of monocyte development [11,12]. The up-regulation of miR-22, -34a and -155 decrease the expression of M-CSFR resulting in the development switch to dendritic cells [13]. However, the overexpression of miR-155 in the hematopoietic compartment causes a myeloproliferative disorder [14]. On the other hand, M-CSF can directly induce PU.1 in HSC, instructing early commitment towards the myeloid lineage [15]. Other regulators of this negative loop are miR-17-5p, miR-20a, and miR106a, as described in detail in [15,16]. Their reduced expression increases the expression of the M-CSF receptor. The overexpression of miR-21 and miR-196b have been shown to promote monopoiesis over granulopoiesis. Both miRNAs are important in the generation of mature monocytes [17]. The role of miR-146a in monopoiesis is not fully understood; however, the overexpression of miR-146a inhibits megakaryopoiesis, and the lack of miR-146a expression in knock-out mice (miR146a^−/−^) results in myeloproliferation [9]. NFκB, a transcription factor involved in the regulation of normal and malignant hematopoiesis, controls the transcription of miR-146a. miR-146a is crucial in regulating monocyte function (see below). A summary of the miRNAs involved in the differentiation of stem cells to monocytes is presented in Figure 1 and Table 1. In cancer, the bone marrow dramatically accelerates the production of monocytes (monopoiesis) [18]. The elevated level of cytokines such as G-CSF, GM-CSF and M-CSF in the serum and low grade of systemic inflammation, which coexists with cancer, contribute to myeloid expansion [19,20]. Monocytes are also produced during cancer progression by extramedullary hematopoiesis [21]; however, only a slight (approximately 2%) support is given from the spleen reservoir [18].

## 3. Monocytes’ Journey in the Blood

The release of monocytes from the bone marrow is regulated by CCR2 and CXCR4 receptors and the signaling associated with them. Monocytes’ exposure to CCL2 (MCP-1) enables their egress by weakening the anchoring of CXCR4 in the bone marrow. Monocytes in bone marrow differ in the level of CXCR4 expression. A higher expression of CXCR4 causes their immobilization in bone marrow [22] and usually correlates with a lower expression of CCR2 [23]. The cross-desensitization model of monocytes’ egress from the bone marrow assumes that under inflammatory conditions the increased availability of CCL2 desensitizes CXCR4 [23,24]. In a mouse model, the expression of CCR2 is regulated indirectly by miR-33 and miR-146 [25,26]. It was shown that CXCR4 expression on mononuclear cells in the bone marrow is regulated by miR-150 [27]. Abrogation of miR-150 by hypoxia significantly increases CXCR4 expression on bone marrow-derived mononuclear cells [27] (Table 1).

The CCR2–CCL2 signaling axis is crucial also for the mobilization of classical monocytes to the tumor site [21,28]. Increased serum level of CCL2 has been observed in patients suffering from different cancers (gastric, pancreatic, prostate, lung) [29,30,31]. These types of cancer have been also associated with an elevated level of circulating monocytes (summarized in [28,32]). The release of monocytes from the spleen during cancer progression is CCR2 independent and is instead associated with angiotensin II signaling [28,33].

Circulating monocytes differ in their CXCR4 expression, which results in different homing preferences. Tumor cells, by secreting TGFβ, upregulate the expression of CXCR4 on monocytes and facilitate their migration towards the CXCL12 gradient [34]. In colorectal cancer, recruited CXCR4^+^ cells are predominantly immunosuppressive (resemble Ly6C^low^) [35], whereas monocytes with lower CXCR4 expression have an enriched profile of genes associated with the innate response [22].

**Table 1:** miRNA in monocytes’ fate.

Monocytes’ Fate		microRNA	Targets	References
**development**		miR-182	C/EBPα	[8]
		miR-125	IRF-4, Bak1, KLF13, BMF	[9]
		miR-34	C/EBPα	[8]
		miR-155	PU.1 aC/EBPα, CSFR1	[9]
		miR-223	NF-1A, E2F1, IKK-1α	[8,9]
		miR-17-5p	RUNX1	[15,16]
		miR-20a	RUNX1	[15,16]
		miR-106a	RUNX1	[15,16]
		miR-21	G-CSF	[17]
		miR-196b	G-CSF	[17]
		miR-146a	TRAF6, IRAK1	[9]
**differentiation**		miR-223	IKKα, E2F1	[36,37]
		miR-17	ATG7	[38]
		miR-22	PU.1	[39]
		miR-106a	RUNX1, CSFR1	[16,40]
**activity**	egress from bone marrow/circulation	miR-19a miR-33 miR-146 miR-150	myosin-IXb, filamin 2, RUNX3 Hmga2, HDL-C CCR2 CXCR4	[41] [25] [26] [27]
	inflammatory response	miR-146a miR-146b miR-132 miR-155 miR-21	NFκB, IRAK1, IRAK2, TRAF6, STAT1, IRF5, Relb STAT3 IRAK4 BCL6, SOCS1, SHIP1 PDCD4	[9,25,26,42] Curtale et al., 2013 [43] [9,43,44,45] [9]
	phagocytosis	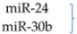 miR-124-5p miR-142-3p miR-21	proinflammatory cytokines ARPC3, ARPC4 PKCα PTEN, PDCD4	[46] [46] [47] [46] [48]

## 4. Monocytes—Diversity and Cancer

**Figure 2:**
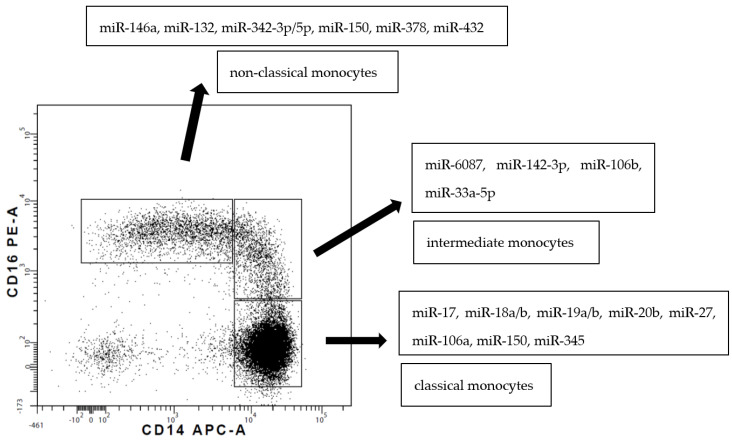
A dot plot representing three subsets of human monocytes according to the expression of CD14 and CD16 supplemented with data concerning upregulated miRNA [41,49,50].

Blood monocytes constitute a heterogeneous population of cells according to their phenotype and function. In humans, circulating monocytes are divided into three subsets, as introduced by Ziegler-Heitbrock et al. in 2010 [5]. The presented review adheres to/is consistent with this nomenclature. The three subsets are described by the gradual change in the expression of CD14 (a lipopolysaccharide co-receptor) and CD16 (an immunoglobulin γ receptor, FcγRIII) markers [5] (Figure 2).

Classical CD14^++^CD16^−^ monocytes represent about 75–85% of the total number of human monocytes and are the counterparts of mouse Ly6C^high^ cells. The subsets are homologous between species, but there are important differences in subset-specific gene expression; thus, these findings cannot be directly translated from mice to humans [51]. Classical monocytes express high levels of CD14 (co-receptor to TLR4, known receptor for LPS), CCR2 and lower levels of HLA-DR. Classical monocytes display high motility towards inflamed tissue and enormous phagocytic potential, which makes them scavenger and rapid reaction force cells [52]. Classical monocytes remain in the blood for roughly 1 day and after that approximately 99% of them leave the circulation [2]. This subset is a major producer of anti-inflammatory IL-10 after endotoxin stimulation [53,54]. The motility of CD16^−^ monocytes is regulated by miR-19a [41]. Prediction analysis has indicated more than 170 target genes involved in cellular movement that might be regulated by miR-19a [41]. The lower susceptibility of the CD16^−^ monocytes to undergo spontaneous apoptosis may be attributed to the low expression of miR-432, which is predicted to regulate a few genes with anti-apoptotic function [41]. Both the decline and elevation of this subpopulation in various malignancies had been observed. The relevant decrease of CD14^++^ monocytes was stated in metastatic melanoma [55] and gastric cancer patients [56]. In contraposition to this report, classical monocytes were meaningfully increased in chronic myelomonocytic leukemia (CMML) [57]. The level of classical monocytes is a potentially predictive marker for anty-PD1 therapy response and patient survival in advanced melanoma [58].

CD14^++^CD16^+^ monocytes are defined as intermediate and, together with the non-classical CD14^+^CD16^++^ subset, are analogous to mouse Ly6C^low^ cells; both are referred to as“proinflammatory” [5]. CD14^+^CD16^++^ cells are described also by CX3CR1^high^ or Slan (6-sulfoLacNAc) expression [59,60]. Intermediate monocytes have a longer lifespan than classical monocytes and circulate in the blood for approximately 4 days [2,3]. Nonclassical monocytes are present in the blood for approximately 7.5 days [2]. CD16^+^ cells patrol the endothelium, transmigrate through a layer of resting endothelial cells and preferentially give rise to dendritic-like cells or macrophages [61,62]. The process of differentiation to dendritic cells is regulated by miR-34 and miR-342, which are expressed abundantly in CD16^+^ monocytes [41]. Functionally, non-classical monocytes are less phagocytic than classical and intermediate monocytes; however, they are extremely important in proinflammatory response (TNF), angiogenesis and the production of ROI (reactive oxygen intermediates) [52]. In the tumor microenvironment, non-classical monocytes are involved in the resolution of inflammation and scavenging tumor-derived materials, e.g., extracellular vesicles [61,63]. The non-classical subset expresses a remarkably high basal level of miR-146a, a known negative regulator of the TLR pathway [26,64]. An elevated level of non-classical monocytes was observed in endometrial or breast cancer patients in comparison with healthy controls. This expansion was associated with a significant increase in CX3CL1 and a reduction in CCL2 levels in cancer patients’ sera [65,66]. An increased ratio of non-classical to CD14^++^ monocytes was observed in patients with multiple myeloma [58]. An increase in the percentage of non-classical and intermediate monocytes was also observed in gastric cancer patients [56]. Additionally, in the cohort study of pediatric patients with solid tumors (neuroblastoma, Wilms’ tumor, retinoblastoma, hepatoblastoma, rhabdomyosarcoma, osteosarcoma, Ewing sarcoma and others) the relevant increase of intermediate and non-classical monocyte subsets was observed and positively correlated with longer overall survival [67]. A significantly elevated percentage of CD16^++^ monocytes was also observed in adult solid cancer patients in comparison to noncancerous, systemically ill patients (e.g., after trauma) [68]. There is also growing evidence implicating non-classical monocytes in the prevention of hematogenic spread of cancer metastasis [63]. Furthermore, a lung cancer mouse model lacking “patrolling” monocytes (Nr4a1 knockout mice) resulted in an increase in metastasis formation [63]. However, elevated level of CD14^+^CD16^++^ monocytes was related to poor prognosis in patients with cholangiocarcinoma [69]. Besides this, a lower survival rate in gastric HER-2-negative patients was correlated with non-classical monocyte tumor infiltration [70]. The conflicting data indicates the divergent roles of non-classical monocytes in different phases of tumor disease.

To date, only a few reports on circulating intermediate subset of monocytes in cancer patients have been published. Intermediate monocytes are major producers of TNF, IL-1β and reactive oxygen species in comparison to the other monocyte subsets [56,71]. Intermediate monocytes exhibit proangiogenic properties, as they express high level of VEGFR2 [72]. An increase of the intermediate subpopulation was observed in patients with ovarian, lung, gastric, colorectal and oral squamous cancer [56,73,74,75,76]. A decreased level of intermediate monocytes was observed in head and neck cancer patients [77]. The confusing and contradictory observations presented above highlight the need to understand the mechanism responsible for the functional heterogeneity of monocytes.

The currently adopted model of the origin of non-classical and intermediate monocytes positions them as a sequential transition from classical monocytes [3]. A recent paper by Tak et al. supported the hypothesis of a linear differentiation pattern from classical monocytes via intermediate to non-classical monocytes [3]. This transition pattern is promoted by serum CCL2 [13]. It was proposed that 1% of classical monocytes end up as circulatory intermediate monocytes [2]. Interestingly, all intermediate monocytes mature in the circulation to become non-classical monocytes [2] Tak at al. assumed that the differentiation of non-classical monocytes from the intermediate subset occurs outside the blood (intermediate cells leave the circulation and return as non-classical monocytes) [3]. It is estimated that about 80–100% of the intermediate subset pool differentiate into non-classical monocytes [2,3]. How classical monocytes are selected to differentiate into non-classical monocytes or how they migrate into the tissue is still unknown. The miR reported to be responsible for regulating the functional heterogeneity of monocyte subsets is miR-146a [26]. RELB, a member of the NF-κB/Rel family, is a direct target of miR-146a. Both, RELB and its negative regulator miR-146a preferentially control the expansion of Ly-6^high^ monocytes in mice (the equivalent of human classical monocytes) [26]. However, recent studies suggest the role of Notch-2 signaling, which promotes the expression of miR-150. miR-150 expression in non-classical monocytes was 10 fold higher than in the classical subset [78]. The major target of miR-150 is TET3. TET3 belongs to the dioxygenase family, which regulates DNA methylation [78]. The orphan transcription factor NR4A1 (nuclear receptor subfamily 4 group A member 1) is required for the differentiation of classical into non-classical monocytes, as investigated in a mouse model [79]. However, as mentioned above, in the mouse model, Ly6C^low^ cells are the counterpart of human non-classical and intermediate monocytes. The latter demonstrated high levels of miR-124-3p [80], which was described as a regulator of NR4A1 [81].

Circulating monocyte subsets differ in miRNA expression. An initial/preliminary study by Dang et al. [41] identified 66 miRNAs that were differentially expressed between CD16^+^ and CD16^−^ monocytes. Targets of differentially expressed miRNAs were mostly related to motility and cell death processes [41]. miRNA-17, miRNA-18a/b, miRNA-19a/b, miRNA-20b, miR-27a, miRNA-106a, miR-119-5b and miR-345 were overexpressed in classical monocytes, whereas miRNA-132, miRNA-146a, miRNA-342-3p, miR-379, miR-382, miR-411, miR-637 and miR-654-3p were overexpressed in non-classical monocytes [50,82] (Figure 2). Recently, the Ziegler-Heitbrock group presented a lower expression of miR-20a and miR-106b in non-classical monocytes defined by both CD14/CD16 expression and via Slan [83].

miRNA analysis of intermediate subpopulations has only been done by Zawada et al. [49]. In this very elegant study, they reported 38 miRNAs that were differentially expressed in the intermediate subset compared to both classical and non-classical monocytes (ibid). Two of them differed in expression more by than 10- and 12-fold: miR-150-5p was downregulated and miR-6087 was upregulated, respectively. Other miRs were linked to distinct biological processes such as cell differentiation, gene regulation, TLRs signaling and antigen presentation. In general, intermediate and non-classical monocytes had the highest similarity in miRNA expression, and the largest differences were found between classical and non-classical monocytes [49].

Shu et al. presented the differentially expressed miRNAs of blood monocytes derived from gastric and breast cancer patients and healthy donors [84]. 74 miRNAs were significantly upregulated in both breast and gastric cancer patients compared with healthy donors, while 46 miRNAs were significantly downregulated in cancers. Most of the target genes of the miRNAs were involved in tumorigenesis including signaling pathways of cancer progression, such as the mTOR signaling, the HIF-1 and the calcium signaling pathways [83].

## 5. Activation of Monocytes—The Role of miRNA

Circulating monocytes are searching for signals that may lead either to their activation, differentiation or death. Inflammatory stimuli such as TLR ligands or proinflammatory cytokines (TNF, IL-1) induce the expression of 200 miRNAs including miR-146a, miR-146b, miR-132 and miR-155 [42]. miR-146a serves as a negative regulator of NFκB by reducing phosphorylation of IκB, which allows its release from the IκB/NFκB complex. miR-146a also targets TRAF6, IRAK1, IRAK2 and IRF3 and controls the TLR4 signaling pathway (Table 1). The enhanced expression of miR-146a limits the inflammatory response of monocytes [42]. The increase of miRNA-146b in monocytes/macrophages may be also mediated by IL-10-dependent STAT3, which may result in the reduction of the LPS-dependent production of inflammatory mediators [85]. Nahid et al. described the induction of miR-132 after TLR2 ligation and hypothesized a feedback regulatory mechanism of miR-132 on the TLR2 signaling cascade, mediated by miR-132 targeting IRAK4 [43]. On the other hand, miR-155 enhances the production of proinflammatory cytokines in monocytes and macrophages and is referred to as a proinflammatory miRNA. The increased expression of miR-155 induced CCL2 secretion by stimulated monocytes/macrophages. It was reported that miR-155 inhibits BCL6, an inhibitor of NFκB [44]. miR-155 activation involves the JNK pathway and results in AKT kinase activation [86].

The role of miRNAs in the regulation of phagocytosis is poorly understood. To date, four miRs have been described as negative regulators of phagocytosis, e.g., miR-24, miR-30b, miR-124-5p and miR-142-3p [46,47]. miR-142-3p directly regulates protein kinase C alpha (PKCα), a key gene involved in phagocytosis. miR-24 and miR-30b regulate the production of TNF-α, IL-6 and IL-12p40, which are associated with active phagocytosis [46]. miR-124-5p is involved in the regulation of the activity of the actin cytoskeleton. The direct targets of miR-124-5p are ARPC3 and ARPC4 transcripts, which result in reduced expression of the ARP2/3 complex, a crucial regulator of actin polymerization [47]. miR-21 was described as directly implicated in switching wound-associated macrophages to an anti-inflammatory status after the engulfment of apoptotic cells or efferocytosis [28]. miR-21 downregulates the inflammatory response via the blocking of PTEN and PDCD4 in TNF-NFκB and IL-10-AP1 pathways [48] (summarized in Table 1).

Cancer cells can alter the activity of circulating monocytes. It has been observed that in cancer patients’ blood, the proportions of monocyte subsets are changed, as described above (“Monocytes—Diversity and Cancer”). Moreover, during tumor development, monocytes with the immunosuppressive phenotype (the downregulation of HLA-DR and upregulation of PD-L1), which are called myeloid-derived suppressor cells (Mo-MDSC), are induced [87]. Finally, monocytes exhibit an altered response to stimuli, e.g., proinflammatory [88] or cancer-related [89]. This process, called “cancer education” or “selective deactivation”, has been described in different types of cancers [89,90]. Cancer forces the differences in the transcriptional profiles of monocytes’ genes, e.g., in breast cancer it applies to 865 genes, and in endometrial cancer to 997 genes. A substantial number of upregulated genes were shown to be involved in cell migration, angiogenesis, cell communication and apoptotic process, as reviewed in [33].

## 6. Macrophage—A Destination of Monocytes

Tissue macrophages are persistent and self-renewing cells [7]. Most of them are established prenatally [7]. However, in adults, tissues are also populated by macrophages derived from bone marrow precursor cells (which could differ from blood monocytes) and by blood monocyte-derived macrophages [7]. Macrophage replenishment is also due to self-renewal [91]. The monocytic origin of tissue macrophages has become a dogma of the mononuclear phagocyte concept, as introduced by Van Furth [92].

For circulating monocytes, cytokines such M-CSF and GM-CSF are the most important signals which trigger their movement into the tissue and differentiation into macrophages or dendritic cells. Under physiological conditions, macrophage homeostasis is regulated by quorum sensing mechanisms in which the M-CSF factor is pivotal. This hypothesis assumes that macrophage survival and proliferation are controlled by the presence of M-CSF in the surroundings and its consumption by them [93]. The homeostatic balance is owed to M-CSF, while GM-CSF is a product of inflammatory activated cells, which contribute to the proinflammatory activation of monocytes [94]. The crucial stage in M-CSF- or GM-CSF-dependent differentiation into macrophages is autophagy, which helps to prevent caspase-3 dependent apoptosis [95,96]. Autophagy in monocytes is ATG7-dependent [97]. The ATG7 gene is negatively regulated by miR-17 [38], which is abundantly expressed in classical monocytes [51]. Another miR involved in the differentiation of monocytes into macrophages is miR-223. The decrease of miRNA-223 causes the repression of IKKα, a component of the NFκB pathway, which then results in the induction of p52 and the repression of both canonical and non-canonical NFκB pathways [36]. In monocytes, miR-223 targets a cell-cycle regulator E2F1, and by blocking the cell-cycle enables differentiation and an exit from the cell cycle [37]. The upregulation of miR-22 in monocytes promotes their differentiation by increasing c-JUN expression and its interaction with PU.1, which controls the whole differentiation process [39]. Macrophages exhibit elevated levels of miR-424-5p, -362-3p, -335-5p and miR-106 in comparison to progenitor cells, which illustrates the role of these miRs in monocytes differentiation [40].

Tumor cells and non-malignant tumor-associated cells (e.g., cancer-associated fibroblasts, stromal cells) secrete a variety of inflammatory factors (IL-6, IL-34, IL-17, M-CSF), chemokines (CCL2, CCL7, CXCL8, CX3CL1) and miRs involved in the recruitment of surrounding macrophages and blood monocytes into the tumor site [40,98]. Breast cancer cells were reported to express high levels of miR-375, which promotes macrophage infiltration via CCL2 [98]. However, the mechanism of how miR-375 enhances CCL2 expression remains unknown. CCL2, in addition to being a strong chemoattractant, can protect monocytes against apoptosis in the tumor microenvironment by upregulating anti-apoptotic proteins and inhibiting caspase-8 cleavage [99]. The overexpression of miR-125 in tumor cells leads to the inhibition of M-CSF and CX3CL1 production by tumors and in consequence the reduction of macrophages recruitment [40,100]. Low levels of miR-148b in hepatocellular carcinoma cells causes the enhanced secretion of M-CSF, which in turn increased macrophages infiltration [40].

Macrophages can be also recruited to the tumor by hypoxia. The differentiation process of blood monocytes (well-oxygenated environment) into macrophages (hypoxic area) changes the expression pattern of hypoxia-inducible factors Hif-1α and Hif-2α, key regulators for the adaptation to hypoxia. This process is associated with a downregulation of the miR-17 -92 cluster, as both Hif-α subunits are targeted by miR-17 and miR-20a [101].

Macrophages that infiltrate the tumor site can adapt their activity to the environment in order to start acting as tumor-associated macrophages (TAM) and to constitute a distinguishable subset of myeloid cells within the tumor stroma. The process by which macrophages produce distinct functional phenotypes in response to environmental stimuli is called polarization [102]. TAM can act as an anti-cancer defender/protector (mostly polarized to M1 or M1-like) but are more likely to support tumor development as M2-polarized macrophages (or M2-like). In many tumors, macrophages of different functional activities may coexist. However, the variety of macrophage subsets makes it hard to find a reliable marker for TAM [103]. In the initial classification, the M1/M2 polarity was based on different metabolic pathways for arginine. M1 macrophages used the iNOS pathway, in contrast to the arginase pathway, are used by M2 cells [102,103]; however, this classification was based on in vitro experiments and does not fully reflect the diversity of macrophages in vivo. In 2008, Mosser and Edwards proposed the new classification of macrophages. They distinguished three major populations, referred to as classically activated (microbicidal activity, mostly similar to M1), wound healing (tissue repair) and regulatory macrophages (anti-inflammatory activity, mostly similar to M2) [104]. The graphical presentation of a color wheel visualized the remarkable plasticity of macrophages, which allows them to change their physiology (color) in response to the environment. Classical macrophages, activated by inflammatory stimuli, e.g., IFN γ and TNF, produce pro-inflammatory cytokines and mediators. Wound-healing macrophages can develop in response to innate or adaptive signals, e.g., IL-4, from injured tissues. The third group of macrophages (regulatory) is generated in response to different stimuli including the combination of TLR ligands, immune complexes or prostaglandins, and is an important producer of TGFβ and IL-10 [104]. Classically activated macrophages with inflammatory phenotypes are dominant in the earliest stages of cancer. During tumor progression, the microenvironment changes the macrophages, which infiltrate the tumor’s neighboring tissue in a way that closely resembles regulatory macrophages. In this classification, TAM share the characteristics of both regulatory and wound healing macrophages and are located in the green area of the color wheel [104].

The polarization of macrophages is regulated at the transcriptional level, e.g., NFkB, STAT1 and C/EBPα mediate M1 polarization by TLR signaling proinflammatory cytokines. IRF4, C/EBPα, KLF4, STAT3 and STAT6 promote M2 polarization [15]. The plasticity of TAMs is regulated, inter alia, by miRNA. miRNAs such as miR-9, miR-21, miR-24, miR-26a, miR-125a, b miR-143, miR-145, miR-146a, miR-148, miR-155, miR-187, miR-223, miR-378-3p, miR-511-3p and others have been implicated in the macrophage polarization process [15]. The short list of miRNAs involved in macrophage polarization is presented below (Table 2).

## 7. Promising Therapies

The progression of various cancers could be enhanced or diminished by the functional activity of macrophages. By regulating macrophage polarization, miRNAs could affect cancer development/outcomes. Although the miRNAs which regulate the polarization of macrophages are known (see above), there is still a lack of clinical trials enabling their utilization.

Among those miRNAs, the most promising studies are associated with miR-155, which could induce the repolarization of M2 to M1 macrophages. In the mouse S-180 sarcoma model, the transfection of miR-155 mimics (double-stranded RNAs, which mimics mature miR-155) to M2 cells induces a switch from M2 to M1 polarized cells. Transduction of pre-miR-155 to M2 macrophages induced apoptosis in Lewis lung carcinoma (LLC) cells [123]. The mechanism is unknown, but some studies suggest that the overexpression of miR-155 leads to the suppression of the C/EBP- β signaling cascade [124]. The potential targets of miR-155 are the SH2-containing inositol-5′phosphatase 1, IL13Rα1 or SMAD2/3 and the suppression of the signaling cascades regulated by these molecules promotes M1 macrophages [45,125,126]. Also, in the pancreatic cancer model, it was presented that exosomes derived from Panc-1 cells transfected with miR-155 or miR-125b-2 resulted in macrophages (J771.A1) reprogramming from the M2 to the M1 phenotype. Transfection was facilitated by hyaluronic acid-poly (ethylene imine; non-viral vector; HA-PEI), which targeted the CD44 molecule on macrophages. Similar results were obtained in the study of lung cancer in mice model using miR-125b [127,128].

Another study revealed that miR-19a-3p is downregulated in M2 macrophages (RAW264.7) in the mouse breast cancer model. The expression of miR-19-3p correlated with the increased expression of the Fra-1 gene (protooncogene) in breast cancer cells [129]. The use of miR-19a-3p mimics significantly suppressed the expression of Fra-1 downstream genes such as VEGF, STAT3 and pSTAT3. In vivo, intratumorally injected miR-19a-3p inhibits the capacity of breast tumor cells (4T1) to migrate and invade. Moreover, miR-19a-3p inhibits the glucocorticoid pathway activating the STAT3 and NFAT and promotes M2 polarization [129].

Some in vitro and in vivo studies showed that miRNA could also control the activity of M-CSF. In 2016, M-CSF was identified as a target of miR-1207-5p in lung cancer cell line A549 [130]. miR-1207-5p contributes to an increase in the secretion of IL-12 and IL-23 and to a decrease in IL-10 and VEGF [110] by regulating STAT3 or AKT kinase. Moreover, this miRNA, via controlling the expression of a few various molecules (e.g., SNAIL, SMAD2 or Vimentin), impacts the regulation of the epithelial–mesenchymal transition (EMT). In the mouse model of lung cancer, the overexpression of miR-1207-5p contributes to the suppression of metastasis formation. In non-small cell lung carcinoma tissues, miR-1207-5p is downregulated and correlates with the upregulated expression of M-CSF [130]. The expression of M-CSF is also regulated by miR-26a. In the hepatocellular carcinoma model (HCC), miR-26a contributes to the stimulation of pro-inflammatory M1 macrophages by downregulating the expression of M-CSF [130]. The elevated expression of M-CSF correlates with a higher frequency of cancer metastasis in many cancer types, e.g., papillary retinal cell carcinomas, breast cancer or HCC. In the vitro model of breast cancer, miR-21 negatively regulates the expression of M-CSF via the regulation of the PI3K/Akt signaling pathway. The inhibition of miR-21 expression by docosahexaenoic acid leads to increased levels of the tumor suppressor protein (PTEN), which prevents the expression of M-CSF [131].

There are also potential anti-tumor therapeutic strategies focusing on the inhibition of autophagy. The autophagic metabolism is characteristic for M2 macrophages and could be potentially useful to modify TAMs polarization. The study presented by Li and collaborators proves the role of miR-498 in the regulation of autophagy in M2 macrophages in esophageal cancer. In this model, the inhibition of MDM2 (mouse double minute 2 homolog)-mediated ATF3 (cyclic AMP-dependent transcription factor) degradation by miR-498 led to both autophagy and the suppression of M2 polarization [132].

Another study showed that diminishing the expression of oncogenic miR-9 in HCC with the use of sponge circMTO1 results in the inhibition of tumor growth. This results in the promotion of the expression of p21, which has a tumor-suppressive role in HCC development [133]. Breast cancer metastasis is promoted by an endogenous noncoding RNA called circIRAK3, which sponges miR-3607. miR-3607 causes the downregulation of FOXC1 (forkhead box C1) [134]. The use of circRNAs that could bind to oncogenic miRNAs and regulate the activity of endogenous circRNAs, which binds to suppressor miRNAs, could be a potential therapeutic strategy; however, further studies are needed to comprehend the mechanisms underlying such regulations.

Another therapeutic strategy against tumors is radiotherapy. However, some tumors could develop resistance to that kind of therapy. In endometrial cancer (EC), resistance was achieved due to exosomes released by infiltrating tumor M2 TAMs. Exosomes carry in abundance circular RNAs (has_circ_0001610). The released has_circ_0001610 absorb/compete with miR-139-5p, causing the upregulation of cyclin B1. These processes lead to the increase of radiotherapy resistance in EC. The knockdown of has_circ_0001610 in in vitro and in vivo results in the increased radiosensitivity of EC. This study showed that knowledge about complicated miRs machinery in macrophages could help to increase cancer sensitivity to therapies, e.g., radiotherapy [135].

## 8. Conclusions

The life span of monocytes and macrophages is controlled by miRs; miRNAs control all stages of monocyte development, activation and differentiation. miRs control macrophage activity in tumors, and changes in the profile of miRNAs may impact the macrophage response to tumor cells by promoting a switch from M2 to M1 cells and/or preventing M2 polarization. Current studies indicate the possibility of using different miRs to ‘control’ the behavior of macrophages and tumor cells. These strategies include a direct interference in M2 to M1 switching (e.g., miR-155) or the regulation of cellular processes such as autophagy (e.g., miR-498), invasiveness (e.g., miR-19a-3p) and growth (e.g., miR-9) in tumor cells themselves. All these examples strongly encourage the exploitation of miRs in clinical trials as good candidates for modern forms of anti-tumor immunotherapy, with the potential of either stimulating an appropriate response from the immune cells infiltrating the tumor or by exerting certain changes in the tumor cells themselves.

## Figures and Tables

**Table 2:** Summary of miRNAs involved in polarization of macrophages.

miRNA	Targets	Mechanism of Action	Ref.
miR-let7c	C/EBP-δ	Promotes M2 by reducing the expression of M1 related genes, e.g., iNOS and IL-12, and increasing levels of M2 markers.	[105,106]
miR-9	NFkB1 PPARδ	Negative regulator of TLR4 signaling, inhibits proinflammatory responses in monocytes/macrophages by suppressing NF-kB1 transcript encoding for the NF-kB subunit p50. miR-9 could also function as a positive regulator of NF-kB signaling by limiting the formation of inhibitory complexes. miR-9 enhances M1 polarization. miR-9 suppresses PPARδ activity and prevents Bcl-6 mediated anti-inflammatory effects.	[15,106,107]
miR-21	M-CSF-R PDCD4	miR-21 suppresses the expression of proinflammatory genes, e.g., iNOS, TNFα and IL-6, and induces the transcription of M2 genes: Arginase1, MRC1, FIZZ and IL-4Rα After induction by LPS, acts as negative regulator of TLR4 signaling by targeting proinflammatory PDCD4, a tumor suppressor.	[9,108]
miR-26a	KLF4 M-CSF PI3K/Akt	The downregulation of miR-26a facilitates the upregulation of KLF4, which increases arginase activity (M2). Its expression leads to the regulation of M-CSF, causing the reduced recruitment of macrophages in HCC (hepatocellular carcinoma).	[108,109]
miR-29-3p	SOCS1/STAT6	Promotes M2 polarization by targeting SOCS1/STAT6, leading to their overexpression.	[110]
miR-124	MCP-1 (CCL2)	Downregulates the expression of CCL2 via direct binding to the 3′UTR of CCL2. The expression of miR-124 is controlled by the expression of ICAM-1, an adhesion molecule on macrophages. Depletion of ICAM-1 leads to M1 polarization because of the lack of CCL2. ICAM-1 induces the expression of the transcription factor Sp1, which regulates miR-124 expression in macrophages.	[111]
miR-125a/b miR-125a-p5	IRF-4 KLF13	Promotes M1 via targeting IRF-4, a negative regulator of the proinflammatory response. The overexpression of miR-125 results in an increased proinflammatory response via, e.g., the enhancing surface expression of MHC II, CD40, CD86, CD80 and IFN-γR. Is involved in the maintenance of M2 macrophages while suppressing the M1 phenotype by KLF13.	[105,112]
miR-127	Bcl-6	Promotes M1 via the targeting of Bcl-6, leading to the limited expression of Dusp1, a negative regulator of JNK activation.	[113]
miR-142-3p	TGFβ	Controls the modulation of macrophages to the M2 phenotype through transforming the growth factor beta (TGFβ) signaling pathway.	[114]
miR-146a	Notch1 TRAF-6 IRAK1 NFkB	Reduces the level of M1-marker genes (e.g., iNOS, CD86, TNF, IL-12 and IL-6), and increases the production of M2-phenotype markers (e.g., Arg1, CCL17, CCL22 and CD206). It is a bona fide negative regulator of NFkB. Suppresses the proinflammatory response and enhances the activation of M2 macrophages via the inhibition of the Notch1 pathway.	[15,85,115]
miR-148a	PTEN SIRPα	Promotes M1 polarization and inhibits M2 polarization upon Notch activation (the reduction of PTEN leads to the activation of AKT and NFkB). miR-148a downregulates the expression of SIRPα (a negative regulator of phagocytosis) on M1 cells.	[116,117]
miR-155-3p miR-155-5p	INPP5D PI3K/AKT SOCS1 SHIP1 TSPAN14 INPP5D MAFB	Promotes inflammation by stopping the expression of INPP5D, an inhibitor of the PI3K/AKT signaling pathway, and SOCS1, which inhibits STATs activity.	[118]
miR-187	MAIL (NFKBIZ) TNFA	Induces the anti-inflammatory response in macrophages by regulating IL-10 secretion. Its overexpression leads to the reduction of the TNFα, IL-6 and IL-12p40 secretion of monocytes activated by LPS.	[15]
miR-223	STAT3	Highly expressed in M2 macrophages. miR-223 overexpression downregulates IL-6 and IL-1b, but not TNF-alpha in TLR-activated macrophages	[119]
miR-375	TNS (tensin3) PXN (paxillin)	Facilitates the recruitment of M2 by acting on CD36. TNS3 and PXN, regulators of cell migration, are direct targets for miR-375. Their downregulation enhances macrophage migration and tumor infiltration.	[98]
miR-378-3p	PI3K/Akt pathway	miR-378 is induced by IL-4 and negatively regulates AKT1 signaling in macrophages. miR-378 promotes M2 polarization by, e.g., the upregulation of Arg1.	[120]
miR-511-3p	CCL2 MRC1 Rock2	Its overexpression leads to the induction of macrophage differentiation to M2 phenotype by reducing the mRNA levels of CCL2. Highly expressed in M2 macrophages and is a coregulator of the mannose receptor expression. By targeting Rock2 (kinase phosphorylating IRF4), miR-511-3p supports the expression of M2-related genes	[121,122]

## Data Availability

Not applicable.

## Abbreviations

Akt	Protein kinase B
ARPC	Actin Related Protein Complex
ATF3	cyclic AMP-dependent transcription factor
ATG7	Autophagy related protein 7
BCL6	B-cell lymphoma 6 protein
C/EBPα	CCAAT/enhancer binding protein α
C/EBP-δ	CCAAT/enhancer binding protein δ
CCR2	c-c chemokine receptor type 2
CD14	a lipopolysaccharide co-receptor
CD16	an immunoglobulin γ receptor, FcγRIII
CMML	chronic myelomonocytic leukemia
CMP	common myeloid progenitor
CSFR1	Colony Stimulating Factor 1 Receptor (M-CSFR)
CX3CL1	c-x3-c motif chemokine ligand 1
CX3CR1	c-x3-c motif chemokine receptor 1
CXCL12 (SDF)	c-x-c motif chemokine ligand 12
E2F1	E2F transcription factor 1
FOXC1	forkhead box C1
GM-CSF	Granulocyte-Macrophage Colony-Stimulating Factor
GMP	Granulocyte-Macrophage Progenitor
HA-PEI	hyaluronic acid-polyethylene imine
HCC	hepatocellular carcinoma
HIF-1	Hypoxia-Inducible Factor 1
HLA-DR	Human Leukocyte Antigen class II
HSC	Hematopoietic Stem Cell
IKKα	IκB kinase α
iNOS	inducible Nitric Oxide Synthase
INPP5D	Inositol Polyphosphate-5-phosphatase D
IRAK1	Interleukin 1 Receptor Associated Kinase 1
IRAK2	Interleukin 1 Receptor Associated Kinase 2
IRAK4	Interleukin 1 Receptor Associated Kinase 4
IRF3	Interferon Regulatory Factor 3
IRF8	Interferon Regulatory Factor 8
JNK	c-Jun N-terminal kinase
KLF4	Kruppel Like Factor 4
KLF13	Kruppel Like Factor 13
LLC	Lewis Lung Carcinoma
LPS	lipopolysaccharide
MAFB	MAF BZIP transcription factor B
MAIL (NFKBIZ)	NFKB inhibitor Zeta
MCP-1 (CCL2)	Monocyte Chemoattractant Protein-1
M-CSF	Macrophage Stimulating Factor
MP	Macrophage Progenitor
MPP	Multipotent Progenitor
MRC1	Mannose Receptor C-type 1
mTOR	mammalian Target of Rapamycin kinase
NFAT	Nuclear Factor of activated T-cells
NFKB1	Nuclear Factor kappa B subunit 1
NFκB	Nuclear Factor kappa-light-chain-enhancer of activated B cells
NR4A1	Nuclear Receptor subfamily 4 group A member 1
PD-1	Programmed cell Death receptor 1
PDCD4	Programmed Cell Death 4 protein
PI3K	Phosphoinositide 3-kinase
PKCα	Protein kinase C alpha
PPAR	Peroxisome Proliferator-Activated Receptor
PTEN	Phosphatase and Tensin homolog deleted on chromosome ten
PU.1	Purine -rich box 1
PXN	paxillin
ROCK2	Rho-associated coiled-coil kinase 2
ROI	Reactive Oxygen Intermediates
RUNX1	Runt-related transcription factor 1
SDF-1 (CXCL12)	α-chemokine receptor specific for stromal-derived-factor-1
SHIP1	src homology 2 domain containing inositol polyphosphate 5-phosphatase 1
SIRPα	Signal regulatory protein α
SOCS1	Suppressor of Cytokine Signaling 1
STAT1	Signal Transducer and Activator of Transcription 1
STAT3	Signal Transducer and Activator of Transcription 3
STAT6	Signal Transducer and Activator of Transcription 6
TAM	Tumor Associated Macrophages
TGFβ	Transforming Growth Factor β
TLR	Toll-like Receptor
TLR2	Toll-like Receptor 2
TLR4	Toll-like Receptor 4
TNF	Tumor Necrosis Factor
TNS	tensin 3
TRAF6	TNF Receptor Associated Factor 6
TSPAN14	tetraspanin 14
VEGFR2	Vascular Endothelial Growth Factor Receptor 2
